# Temporal changes of *Sall4* lineage contribution in developing embryos and the contribution of *Sall4*-lineages to postnatal germ cells in mice

**DOI:** 10.1038/s41598-018-34745-5

**Published:** 2018-11-06

**Authors:** Naoyuki Tahara, Hiroko Kawakami, Teng Zhang, David Zarkower, Yasuhiko Kawakami

**Affiliations:** 1 Department of Genetics, Cell Biology and Development, University of Minnesota, Minneapolis, MN USA; 2 Stem Cell Institute, University of Minnesota, Minneapolis, MN USA; 3 Developmental Biology Center, University of Minnesota, Minneapolis, MN USA

## Abstract

Mutations in the *SALL4* gene cause human syndromes with defects in multiple organs. *Sall4* expression declines rapidly in post-gastrulation mouse embryos, and our understanding of the requirement of *Sall4* in animal development is still limited. To assess the contributions of *Sall4* expressing cells to developing mouse embryos, we monitored temporal changes of the contribution of *Sall4* lineages using a *Sall4 GFP-CreER*^*T2*^ knock-in mouse line and recombination-dependent reporter lines. By administering tamoxifen at various time points we observed that the contributions of *Sall4* lineages to the axial level were rapidly restricted from the entire body to the posterior part of the body. The contribution to forelimbs, hindlimbs, craniofacial structures and external genitalia also declined after gastrulation with different temporal dynamics. We also detected *Sall4* lineage contributions to the extra-embryonic tissues, such as the yolk sac and umbilical cord, in a temporal manner. These *Sall4* lineage contributions provide insights into potential roles of *Sall4* during mammalian embryonic development. In postnatal males, long-term lineage tracing detected *Sall4* lineage contributions to the spermatogonial stem cell pool during spermatogenesis. The *Sall4 GFP-CreER*^*T2*^ line can serve as a tool to monitor spatial-temporal contributions of *Sall4* lineages as well as to perform gene manipulations in *Sall4*-expressing lineages.

## Introduction

*Sall4* is one of four *Sall* genes that encode zinc finger transcription factors^1,2^. Heterozygous mutations in the human *SALL4* gene cause Duane-radial ray syndrome (also known as Okihiro syndrome), an autosomal dominant disorder^3,4^. *SALL4* mutations are also found in Acro-renal-ocular syndrome^5,6^. It is considered that these syndromes are caused by *SALL4* haploinsufficiency^3^. The patients exhibit upper limb deformities and aberrant ocular movements due to defects in specific nerves. Other symptoms include renal agenesis, unilateral deafness, choanal atresia, external ear malformations, and ventricular septal defect with varying degrees^3,7^. The radial ray malformations are observed in the anterior forelimbs and include hypoplasia or aplasia of the thumbs and/or the radius, triphalangeal thumbs, and preaxial polydactyly.

Heterozygous *Sall4* mutant mouse phenotypes partially recapitulate human patients’ symptoms. For instance, *Sall4*^+/−^ mice exhibit anal stenosis, ventricular septum defects, exencephaly, hypoplastic kidney, anogenital tract abnormalities, conductive deafness, neural tube closure defects and kinky tails with different penetrance of phenotypes^8–10^. In contrast, limb defects were not observed in *Sall4*^+/−^ mice. The human patient symptoms and *Sall4*^+/−^ mouse phenotypes together indicate that SALL4 plays important roles in the development and function of a variety of tissues and organs and that many of these functions are likely to be conserved among mammals. However, our understanding of *Sall4* functions during mammalian development remains incomplete, mainly due to the peri-implantation lethality of *Sall4* null mouse embryos, which has hampered analysis of *Sall4* post-implantation functions^8,11^.

The *Sall4* expression pattern during mouse embryonic development provides insights into likely *Sall4* functions during mammalian development. In pre-implantation stages, SALL4 protein is detected in the two-cell stage embryos due to maternal contribution^11,12^. After zygotic gene expression starts, *Sall4* transcripts are detected in early cleavage stages^10–12^. In blastocysts, *Sall4* is expressed in the inner cell mass and trophoectoderm^8,11^. During these stages *Sall4* activity contributes to proliferation of cells in the inner cell mass of blastocysts^8^. In addition, a recent report using single cell technology demonstrated that *Sall4* modulates gene regulatory networks to promote commitment of inner cell mass cells to pluripotent epiblast or primitive endoderm^13^.

After implantation, *Sall4* is expressed uniformly in the epiblast until mid-streak stages (~embryonic day (E) 6.5)^11^. *Sall4* null embryos arrest around the peri-implantation stage^8,11^, indicating its critical role in the epiblast during this period. *Sall4* is widely expressed in E7.5 embryos, and strong expression is gradually confined to the head and primitive streak while weaker expression is maintained broadly in E8.5 embryos^14^. After completion of gastrulation, *Sall4* is highly expressed in the posterior body, such as the tail bud and in the presomitic mesoderm, the un-segmented posterior paraxial mesoderm, at E9.5–E12.5^14,15^. During these stages, *Sall4* expression in most other areas of embryos rapidly declines and expression becomes confined to small domains.

In addition to the tail bud, the limb bud is one of the *Sall4*-expressing regions in the post-gastrulation stages. During limb development, *Sall4* expression is detected in the mesenchyme from the beginning of outgrowth in both forelimb and hindlimb buds^14–16^. *Sall4* expression is confined to the distal mesenchyme by E10.5, becomes anteriorly biased at E11.5, and is confined to the narrow, distal-most region by E12.5.

Other than tail and limb buds, *Sall4* is expressed in craniofacial structures^14,16^. *Sall4* is expressed in the frontonasal mesenchyme, the midbrain, the mandibular arch and maxillary arch at E9.5–E10.5. At E11.5–E12.5, *Sall4* expression rapidly declines but remains detectable in the midbrain. *Sall4* is also expressed in the developing genital tubercle at E11.5–E12.5^14^.

In postnatal mice, *Sall4* is expressed in undifferentiated spermatogonia in the testis^17–19^. Functional analysis showed that *Sall4* is essential for maintenance of undifferentiated spermatogonia^20^. In female germ cells, *Sall4* is highly expressed in oocytes at different developmental stages^12^. Deletion of *Sall4* in the primary follicle stage or primordial follicle stage oocytes causes oocyte immaturity and infertility, demonstrating the requirement for *Sall4* in female germ cells.

These expression patterns suggest that *Sall4* plays roles in various tissues and organs during embryonic development and in germ cells. As described above, however, the functions of *Sall4* in tissue/organ development remain largely unknown in post-implantation mouse embryos due to the early lethality of *Sall4* null mutants^8–11^. Previous studies of *Sall4* expression patterns have provided some insights into probable *Sall4* functions during post-gastrulation mouse development^14–16^. However, because *Sall4* expression declines rapidly in post-gastrulation stages in mouse embryos it is likely that *Sall4*-expressing cells dynamically change their contributions to developing tissues and organs in mouse embryos. Therefore, we sought to gain insights into the roles of *Sall4* by determining the contributions of *Sall4*-expressing cells in early post-gastrulation stages through mid-gestation stage mouse embryos. For this purpose, we performed genetic tracing of *Sall4*-expressing cells using an inducible Cre cassette knocked into the *Sall4* 1^st^ exon together with recombination-dependent reporter mouse lines that monitor *Sall4*-*Cre* activity.

## Results

### The GFP signals of *Sall4 GCE* knockin mice reflect high levels of *Sall4 mRNA* expression

**Figure 1:**
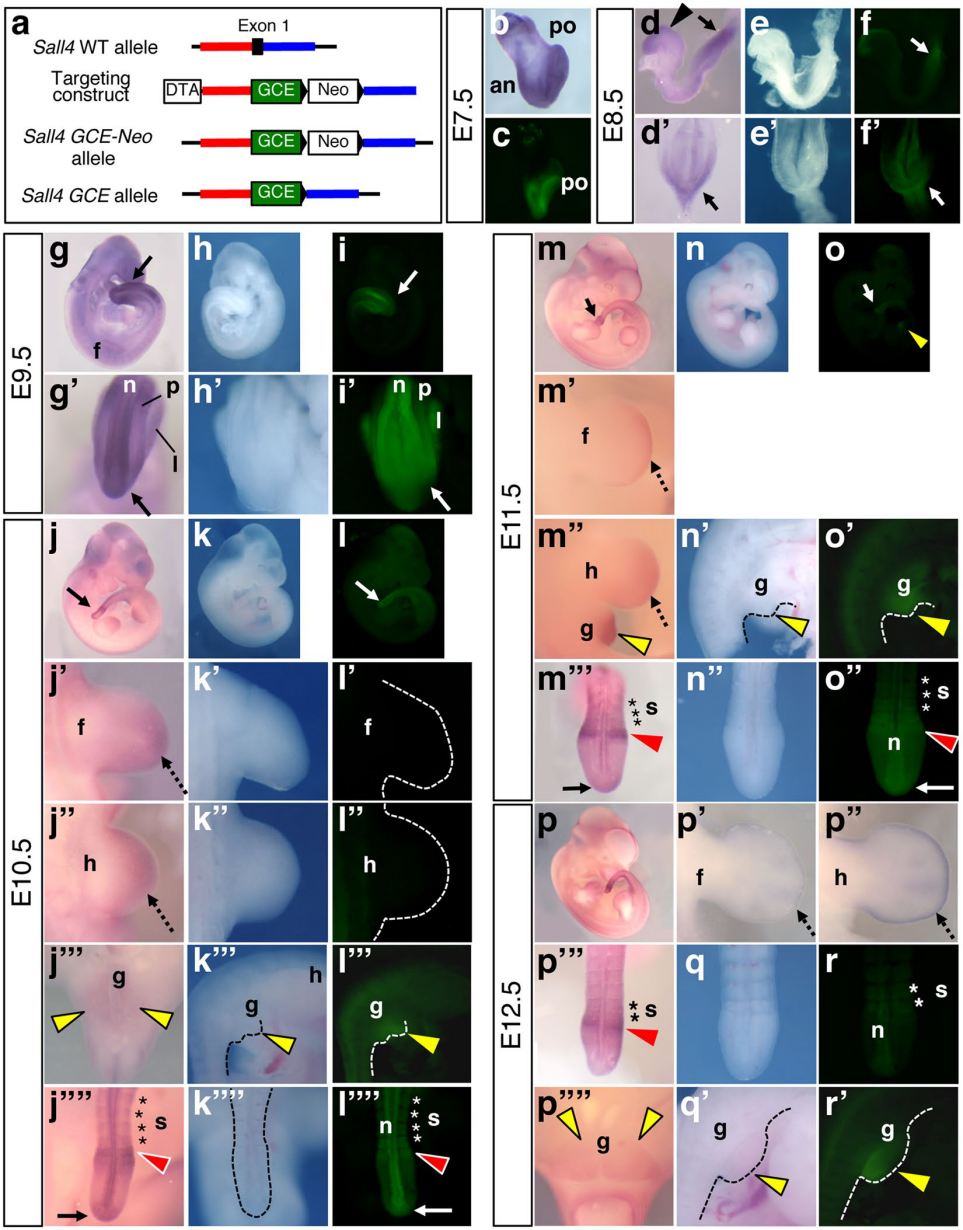
The GFP signals of *Sall4 GCE* embryos were detectable in cells/tissues with high levels of *Sall4* mRNA expression. (**a**) Schematic of targeting strategy to knock-in the *GCE* cassette into the exon 1 of the *Sall4* gene. (**b**,**d**,d’,**g**,g’,**j**–j””,**m**–m’”,**p**–p””) *Sall4* mRNA expression pattern of indicated stages by whole mount *in situ* hybridization. Bright field images (**e**,e’,**h**,h’,**k**–k””,**n**–n”,**q**,q’) and the GPF images (**f**,f’,**i**,i’,**l**–l””,**o**–o”,**r**,r’) of *Sall4 GCE* embryos at indicated stages. At E8.5 (**d**–f’) and E9.5 (**g**–i’), arrowheads and arrows point to the head and the posterior tip of the body. Panels in b,c,d,e,f,g,h and i show lateral views of the whole embryo. Panels in d’,e’,f’,g’,h’ and i’ show dorsal views of the posterior part of the body. At E10.5–E12.5, dashed black arrows point to the signal at the distal part of the limb buds in dorsal views with the anterior to the top (j’,j”,m’,m”,p’,p”). Arrows point to the posterior tip of the tail (**j**,j””,**l**,l””,**m**,m”’,**o**,o”). Red arrowheads point to the anterior presomitic mesoderm in dorsal views (j””,l””,m’”,o”,p’”). Yellow arrowheads point to the external genital primordium (j’”,k’”,l’”,m”,n’,**o**,o’,p””,q’,r’). The genital primordia are shown in the ventral views (j’”,p””) or lateral views (k’”,l’”,m”,n’,o’,q’,r’). Asterisks in j””,l””,m’”,o”,p’” and **r** indicate signals in the somites. Abbreviations. an: anterior, f: forelimb bud, g: external genital primordium, h: hindlimb bud, l: lateral plate mesoderm, n: neural tube, p: paraxial mesoderm, po: posterior, s: somites.

To detect contribution of *Sall4* expressing cells at a given time of development, we generated a novel allele, in which *GFP-CreER*^*T2*^ (*GCE*) is knocked into the 1^st^ exon of the *Sall4* gene (Fig. 1a). Loss of *Sall4* leads to embryonic lethality^8–11^ and as expected *Sall4*^*GCE/GCE*^ homozygous mutant pups were not recovered from crosses of heterozygotes (not shown). We first compared *Sall4* mRNA expression pattern and the GFP signals at E7.5 – E12.5. At E7.5, both *Sall4* mRNA and the GFP signals were broadly detected (Fig. 1b,c). At E8.5, the GFP signals were readily detectable only in the anterior and posterior parts of the body (Fig. 1f,f’). At this stage, *Sall4* is broadly expressed, and high levels of *Sall4* mRNA expression are confined to the anterior and posterior parts of the body (Fig. 1d,d’). The GFP signals were not detected in tissues with weaker *Sall4* mRNA signals. At E9.5, strong GFP signals were detected in the posterior part of the body (Fig. 1i,i’), where strong *Sall4* mRNA signals were also detected (Fig. 1g,g’). Higher magnification images show that signals were in the posterior neural plate and paraxial and lateral plate mesoderm tissues (Fig. 1g’,i’). At E10.5, the GFP signals were detected in the posterior tip of the tail bud, posterior neural tube, anterior presomitic mesoderm and somites (Fig. 1l,l””), where *Sall4* mRNA is expressed at high levels (Fig. 1j,j””). The GFP signals and *Sall4* mRNA were also detected in the genital primordium (Fig. 1j’”,l’”). Although *Sall4* mRNA is expressed in the distal portions of forelimb and hindlimb buds (Fig. 1j’,j”)^14–16^, the GFP signals were undetectable in limb buds at E10.5 (Fig. 1l’,l”). At E11.5, the GFP signals were detected in the tail bud, presomitic mesoderm and somites (Fig. 1o,o”), consistent with strong expression of *Sall4* mRNA in the tail bud and anterior presomitic mesoderm (Fig. 1m,m’”). The GFP signal and *Sall4* mRNA were also detected in the genital primordium (Fig. 1m”,o’). Similar to E10.5 embryos, the GFP signals were undetectable in limb buds, although *Sall4* mRNA is expressed (Fig. 1m’,m”). At E12.5, *Sall4* mRNA is expressed in the anterior presomitic mesoderm, while the expression is significantly reduced in the tail bud tip and distal-most limb mesenchyme (Fig. 1p-p’”). At this stage, weak GFP signals were detected in the posterior somites (Fig. 1r). Strong GFP signals were maintained in the genital primordium (Fig. 1r’), consistent with *Sall4* mRNA expression (Fig. 1p””).

These signal comparisons indicate that the GFP signals in *Sall4 GCE* embryos are highly congruent with endogenous *Sall4* mRNA expression but detectable only in cells/tissues where high levels of *Sall4* mRNA are expressed. In particular, the GFP signals were consistently detectable in the posterior tissues (tail bud, neural tube, presomitic mesoderm) and the external genitalia. In contrast, the GFP signals became undetectable during the early outgrowth stages of the limb bud.

### The *Sall4*-lineage contribution is rapidly restricted to the posterior of the body in post-gastrulation stages

**Figure 2:**
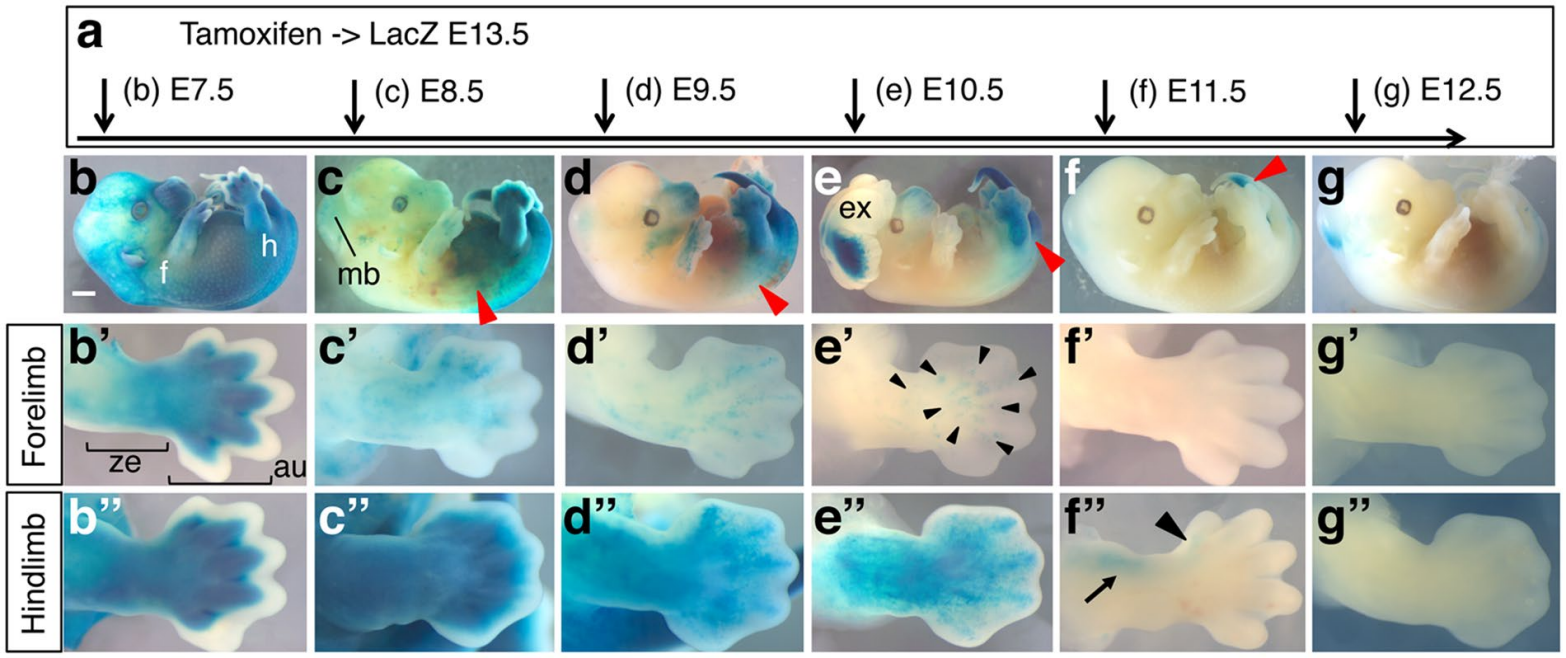
*Sall4* lineage contribution to E13.5 embryos. Schematic of tamoxifen injections at different time points of embryonic development. The stages of tamoxifen injection are indicated. (**b**–**g**) Whole mount images of LacZ-stained E13.5 embryos. Red arrowheads in c-g point to the anterior margin of LacZ staining. The embryo in panel **e** exhibits exencephaly. (b’–g’) Dorsal views of forelimbs of embryos shown in (**b**–**g**). Black arrowheads in **e**’ point to sparsely stained LacZ-positive areas. In panel b’, the autopod region (au) and the zeugopod region (ze) are indicated by brackets. (b”–g”) Dorsal views of hindlimbs of embryos shown in (**b**–**g**). Black arrowhead and arrow in f” point to LacZ-positive anterior digit and anterior zeugopod, respectively. Anterior is to the top and posterior is to the bottom in b’–g’ and b”–g”. Abbreviations: au: autopod region, fl: forelimbs, hl: hindlimbs, ze: zeugopod region. Scale bar in panel b: 1 mm. Panels b to g are in the same scale.

To compare contributions of *Sall4*-expressing cells at different stages, we crossed *Sall4 GCE* males with *R26-LacZ* females, and administered tamoxifen to the pregnant females at different time points. Embryos were then collected and stained for LacZ activities at E13.5 (Fig. 2a). Consistent with the broad expression of *Sall4* mRNA and GFP from the *GCE* allele at E7.5 (Fig. 1b,c), tamoxifen injection at E7.5 resulted in essentially ubiquitous LacZ staining (Fig. 2b). LacZ-labelled *Sall4* lineage contribution decreased in the anterior of the body by E8.5, with staining detected mainly posterior to the middle of the trunk (Fig. 2c). The anterior border of the *Sall4* lineage contribution was anterior to the hindlimb when tamoxifen injection was performed at E9.5 (Fig. 2d), while injection at E10.5 shifted the strong LacZ staining domain posterior to the hindlimb, with weaker LacZ staining detected in the flank immediately anterior to the hindlimb (Fig. 2e). The contribution became restricted to the tail tip by E11.5 (Fig. 2f) and was undetectable in the tail at E12.5 (Fig. 2g). These results show a rapid and spatially dynamic reduction of *Sall4* lineage contribution to the posterior of the body during E8.5–E11.5. It has been reported that *Sall4*^+/−^ embryos exhibit exencephaly at low penetrance^8^. Because the exon 1 is replaced with the *GCE* cassette in the *Sall4 GCE* allele (Fig. 1a), we also observed low penetrance exencephaly in *Sall4 GCE; R26-LacZ* embryos, as expected if the *GCE* allele is null or severely hypomorphic (Fig. 2e).

### Reduction of *Sall4* lineage contributions is different in forelimbs and hindlimbs

The limb develops from specific regions of lateral plate mesoderm, arising from the 7–12 somite levels for forelimbs and the 25–29 somite levels for hindlimbs. We found that *Sall4* lineages at E7.5, before formation of the limb-forming regions, contribute to both forelimbs and hindlimbs (Fig. 2b’,b”). At E8.5 (8–12 somite stage), the forelimb-forming region is specified but the hindlimb-forming region has not been established. At this stage we found that the *Sall4* lineage sparsely contributed to forelimbs with more contributions to the anterior portion (Fig. 2c’). In contrast precursors of hindlimbs are still in the posterior lateral plate mesoderm at this stage, and *Sall4* mRNA is highly expressed in this region. Similarly, *Sall4* lineages at E8.5 broadly contributed to the hindlimbs (Fig. 2c”). At E9.5 (20–24 somite stage), forelimb buds have been formed, but hindlimb buds are just initiating outgrowth in the posterior region of the elongating body. The contribution of *Sall4* expressing cells to forelimbs at this stage was detected in a sparse and anteriorly biased manner, and the contribution was reduced relative to E8.5 (Fig. 2d’). The contribution to hindlimbs was still broadly detected, also with less LacZ signal intensity than at E8.5 (Fig. 2d”). At E10.5, both forelimb buds and hindlimb buds are developed, and *Sall4* mRNA is expressed in both types of limb buds. *Sall4* contribution from the *GCE* allele was detected in a very sparse manner in the autopod of forelimbs (Fig. 2e’), but the contribution to hindlimbs was still broadly detected (Fig. 2e”). At E11.5 and E12.5, *Sall4* lineage contributions to forelimbs were not detected (Fig. 2f’,g’), although *Sall4* mRNA is expressed at these stages (Fig. 1m’,p’)^14,16^. *Sall4* lineage contribution at E11.5 to hindlimbs was detected in the anterior zeugopod and the most anterior digit (Fig. 2f”). *Sall4* contributions to both forelimbs and hindlimbs became undetectable at E12.5 (Fig. 2g’,g”).

In summary, in both forelimbs and hindlimbs, *Sall4* lineages contributed broadly to the limb when tamoxifen was injected before specification of limb progenitors (Fig. 2b’,b”,c”). When tamoxifen was injected around the time of specification of forelimb progenitors (~E8.5) and hindlimb progenitors (~E9.5), *Sall4* lineage contribution to the limb declined. Even with such similarities, *Sall4* lineage contribution to hindlimbs persisted longer than that to forelimbs.

### *Sall4* lineage contribution to the craniofacial structures

**Figure 3:**
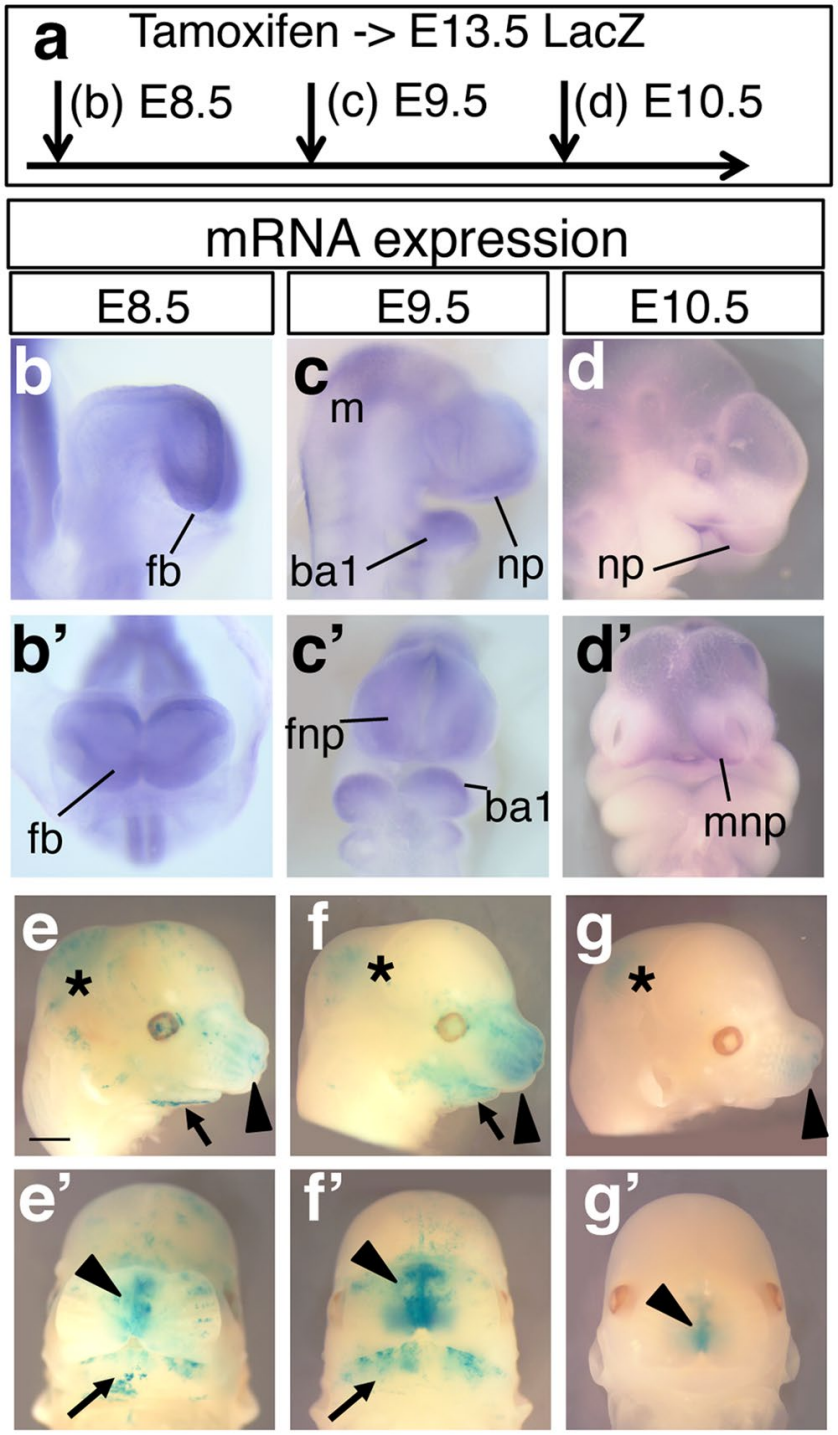
*Sall4* lineage contribution to the craniofacial structures. Schematic of tamoxifen injections at different time points of embryonic development. (**b**–d’) Lateral views (**b**–**d**) and frontal views (b’–d’) of *Sall4* mRNA expression at indicated stages. (**e**–g’) Lateral views (**e**–**g**) and frontal views (e’–g’) of LacZ stained E13.5 embryos. Asterisks indicate LacZ signals in the midbrain. Black arrowheads and arrows point to signals in the nasal region and the lower jaw, respectively. Abbreviations: ba1: branchial arch 1, fb: forebrain, fnp: front nasal process, m: midbrain, mnp: medial nasal process, np: nasal process. Scale bar in panel e: 1 mm. Panels e to g’ are in the same scale.

*Sall4* is expressed in craniofacial structures. At E8.5, *Sall4* mRNA is expressed in the forebrain (Fig. 3b,b’), and *Sall4* lineage contribution at E8.5 was detected in the nasal structure and lower jaw (Fig. 3e,e’). At E9.5, *Sall4* mRNA expression is evident in the midbrain, nasal process and the first branchial arch (Fig. 3c,c’). *Sall4* lineage contribution at E9.5 was detected as slightly broader than that at E8.5, and the LacZ signal was more intense than at E8.5 (Fig. 3e,e’,f,f’). At E10.5, *Sall4* mRNA is expressed in the front nasal process (Fig. 3d,d’). *Sall4* lineage contribution at this stage was detected in the distal tip of the nasal structure (Fig. 3g,g’) and more weakly in the midbrain. After E11.5, *Sall4* lineage contribution was not detected in the nasal structure (Fig. 2f,g).

These results show that *Sall4* lineage contribution to the craniofacial structures becomes slightly broader from E8.5 to E9.5; however, between E9.5 and E10.5, the contribution declines rapidly, similar to the contribution to the axial level.

### *Sall4* lineage contribution to the external genitalia

**Figure 4:**
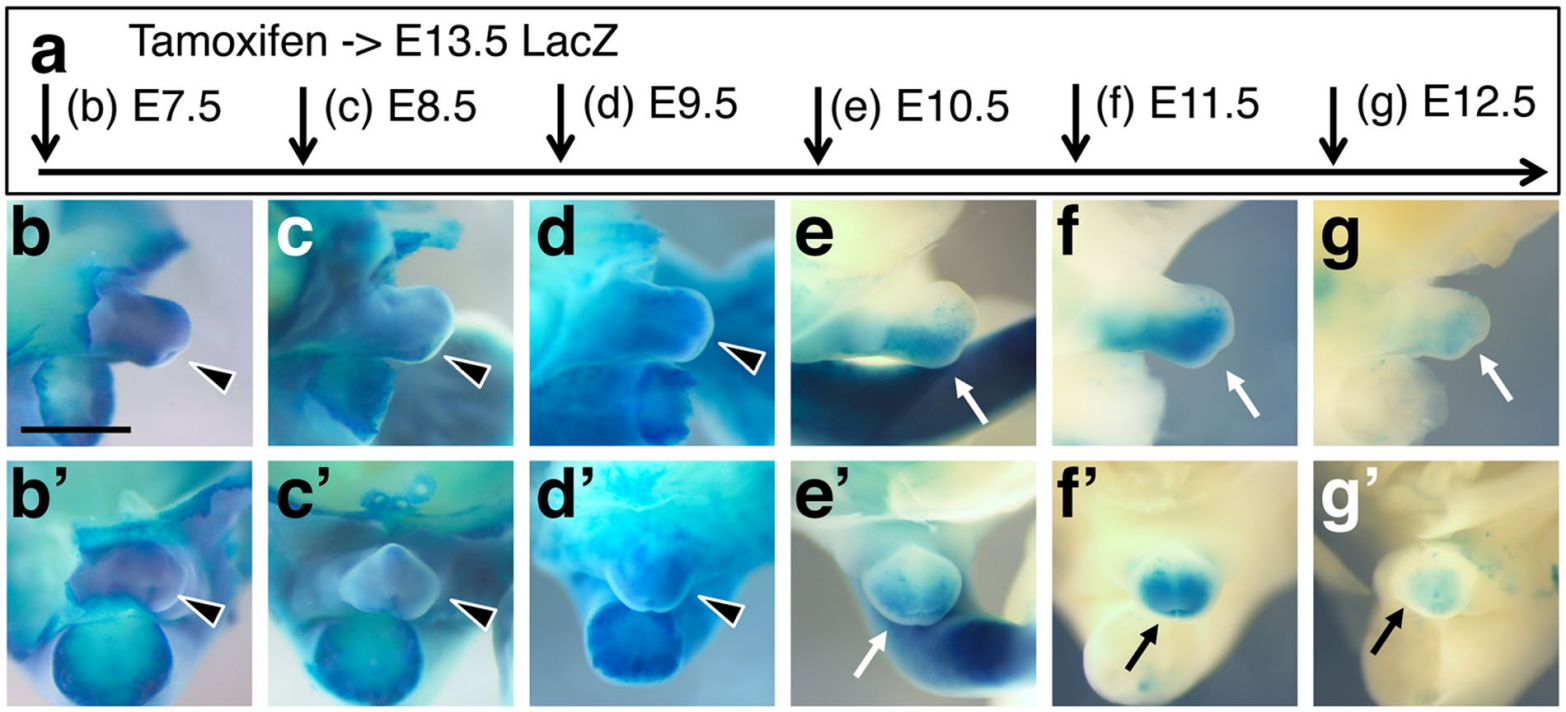
*Sall4* lineage contribution to the external genital primordium. Schematic of tamoxifen injections at different time points of embryonic development. (**b**–g’) Lateral views (**b**–**g**) and frontal views (b’–g’) of external genitalia of LacZ stained E13.5 embryos. Black arrowheads point to the broadly stained external genitalia, labelled at E7.5–E9.5. Arrows point to the LacZ-stained posterior of external genitalia, labeled at E10.5–12.5. Scale bar in panel b: 1 mm. Panels b to g’ are in the same scale.

While *Sall4* mRNA expression rapidly declines in post-gastrulation stages, the external genital primordium remains a strong *Sall4* expressing domain (Fig. 1j’”,l’”,m”,o’,p””,r’). During E7.5 – E9.5, when *Sall4* lineages contribute to the posterior body (Fig. 2b,c,d), *Sall4* lineages were detected in the entire external genitalia (Fig. 4b,b’,c,c’,d,d’). In contrast, *Sall4* lineage contribution was restricted to the posterior half of the genital primordia at E10.5 and E11.5 (Fig. 4e,e’,f,f’). At E12.5 *Sall4* lineage contribution was still detected in the posterior of external genitalia, but with reduced signal intensities (Fig. 4g,g’).

### The *Sall4* lineage contributes to the extra-embryonic tissues, such as yolk sac and umbilical cord

**Figure 5:**
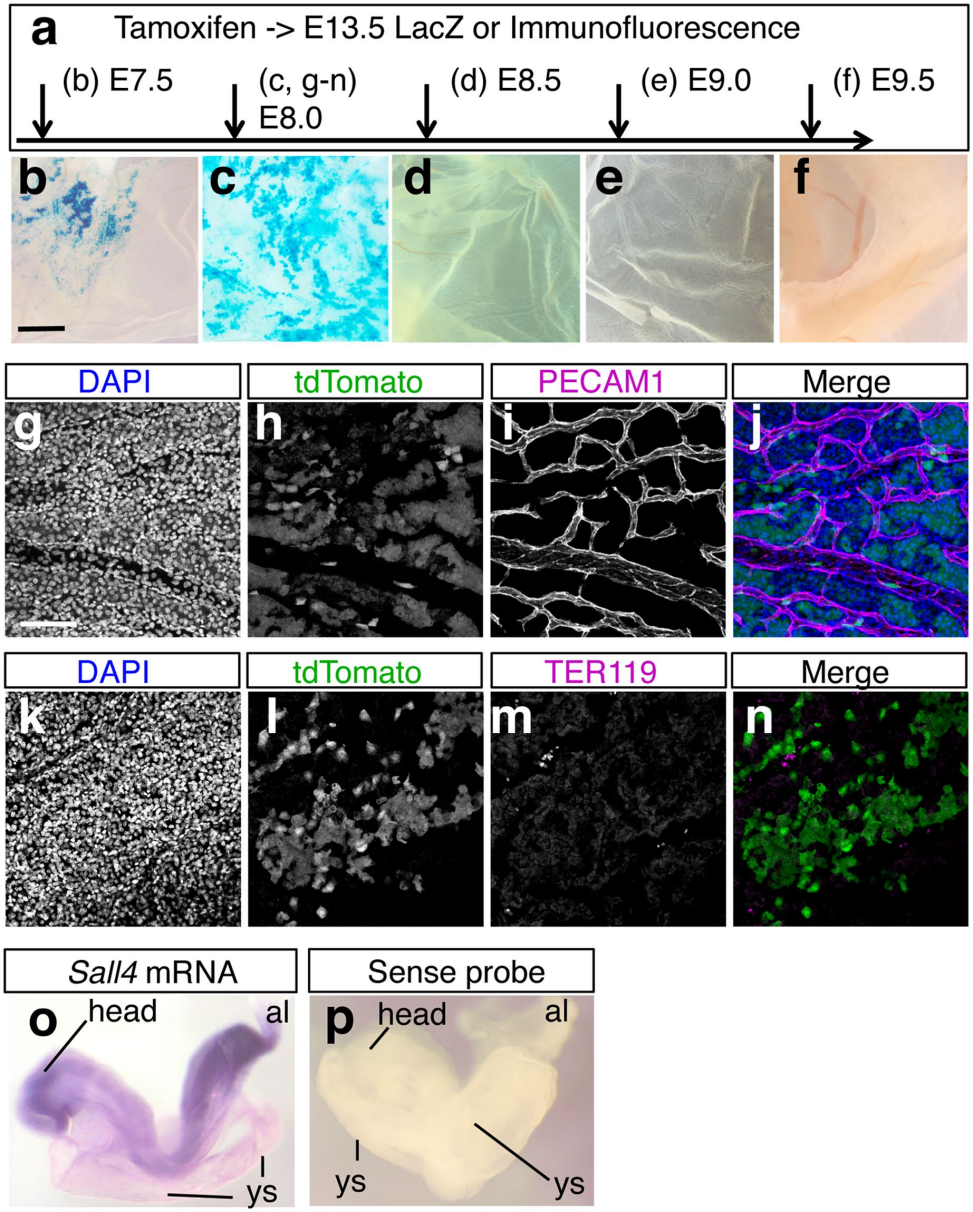
*Sall4* lineage contribution to the yolk sac stroma in a narrow time window. Schematic of tamoxifen injections at different time points of embryonic development. (**b**–**f**) LacZ-stained yolk sac. The staining was broadly detected when tamoxifen was injected at E8.0. Scale bar in panel b: 1 mm. Panels b to f are in the same scale. (**g**–**n**) Immunofluorescence images of DAPI (**g**,**k**), tdTomato (**h**,**l**), PECAM (**i**) and TER119 (**m**). (**j**) and (**n**) show merged images of (**g**–**i**) and (**k**–**m**), respectively. The tdTomato signals do not overlap with PECAM and TER119. Scale bar in panel g: 100 µm. Panels g to n are in the same scale. (**o**,**p**) Lateral views of E8.5 embryos hybridized with antisense (**o**) or sense (**p**) *Sall4* probes. *Sall4* is expressed in the yolk sac (ys) and the allantois (al), in addition to the embryo. Control sense probe generated no signals. Abbreviations. al: allantois, ys: yolk sac.

*Sall4* expression in extra-embryonic tissues has not been reported. Unexpectedly, we found that *Sall4* lineages contribute to extra-embryonic tissues such as the yolk sac in a very transient manner. Labeling *Sall4* lineages at E7.5 resulted in patchy LacZ staining in the yolk sac (Fig. 5b). When tamoxifen injection was performed at E8.0, LacZ staining was broadly detected in the yolk sac (Fig. 5c). However, tamoxifen injection at E8.5, E9.0 and E9.5 resulted in little LacZ signal (Fig. 5d,e,f). This staining pattern demonstrates a very narrow time window during which *Sall4* lineages contribute to the yolk sac.

To determine whether *Sall4* lineages contribute to hematopoietic or endothelial cells in the yolk sac at E13.5, we performed immunofluorescence analysis. For this purpose, we used *R26-tdTomato*, instead of *R26-LacZ*, and co-detected tdTomato by mCherry antibodies together with anti-PECAM1 (endothelial marker, Fig. 5i) or anti-TER119 (blood cell marker, Fig. 5m). Fluorescent images showed that tdTomato-positive *Sall4* lineages did not overlap with PECAM1 (Fig. 5g–j) and TER119 (Fig. 5k–n). These results indicate that *Sall4* lineages contribute to the yolk sac stroma during a very narrow time window around E8.0.

To determine the origin of the contribution of *Sall4* lineages to the extra-embryonic tissues, we re-examined *Sall4* expression by *in situ* hybridization. Due to a lag from tamoxifen injection to CreER-dependent recombination^21^ (see Discussion), we used E8.5 embryos and detected *Sall4* expression in the yolk sac and allantois (Fig. 5o,p). The expression pattern supports the idea that *Sall4* expressing cells in the yolk sac contribute to the yolk sac stroma at later stages.

### The *Sall4* lineage contributes to the peri-vascular tissues in the umbilical cord

**Figure 6:**
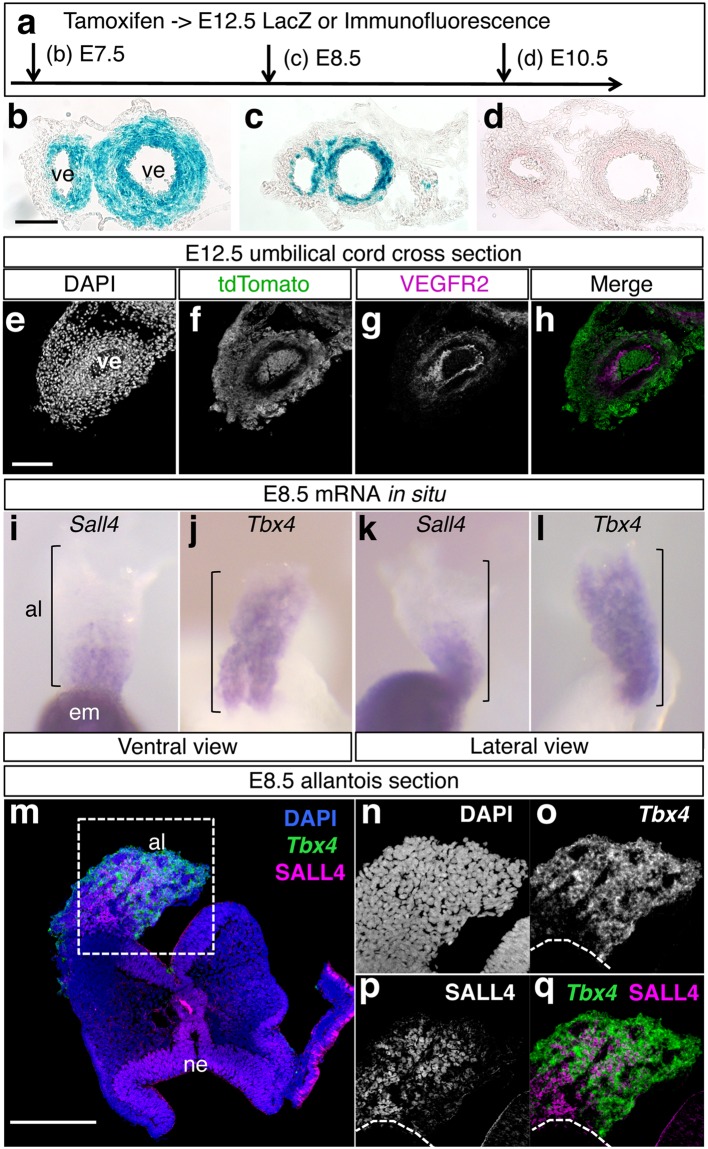
*Sall4* lineage contribution to peri-vascular mesenchyme in the umbilical cord. Schematic of tamoxifen injections at different time points of embryonic development. (**b**–**d**) Cross section of LacZ-stained umbilical cord. Scale bar in panel b: 100 µm. Panels b to d are in the same scale. (**e**–**h**) Immunofluorescence images of DAPI (**e**), tdTomato (**f**), VEGFR2 (**g**) and merged image (**h**). The tdTomato signals do not overlap with VEGFR2. Scale bar in panel e: 100 µm. Panels e to h are in the same scale. (**i**–**l**) Whole mount *in situ* hybridization of *Sall4* (**i**,**k**) and *Tbx4* (**j**,**l**) of E8.5 embryos. Ventral views (**i**,**j**) and lateral views (**k**,**l**) of the allantois are shown. (**m**–**q**) Double detection of SALL4 immunoreactivities (magenta) and *Tbx4* mRNA (green) on allantois sections. Panels n–q show closeup of the dotted square in (**m**). Panels n, o and p are shown in a black/white mode. Dotted lines in (**o**–**q**) indicate the border between the allantois and the embryo. Scale bar in m: 200 µm. Abbreviation. al: allantois, em: embryo, ne: neuroectoderm of the head region, ve: vessel.

The allantois is the precursor tissue of the umbilical cord^22^. Expression of *Sall4* in the allantois, found in this study (Fig. 5o,p) suggests that *Sall4*-expressing cells contribute to umbilical cord. Therefore, we examined the *Sall4* lineage contribution to the umbilical cord by sectioning LacZ-stained E12.5 umbilical cord. We found that tamoxifen injection at E7.5 resulted in broad LacZ signals around the two vessels of the umbilical cord (Fig. 6b). When tamoxifen injection was done at E8.5, *Sall4* lineages contributed less to the tissue around the vessels (Fig. 6c). No contribution to tissues around the umbilical vessels was detectable when tamoxifen was administered at E10.5 (Fig. 6d). Immunofluorescence of cross sections of the umbilical cord at E13.5, labelled at E8.0, showed that tdTomato-positive *Sall4*-lineage did not overlap with VEGFR2, a vascular endothelial cell marker (Fig. 6e–h). These results support the idea that the *Sall4* lineage transiently contributes to the peri-vascular mesenchyme in the umbilical cord.

A previous lineage tracing experiment demonstrated that *Tbx4*-expressing cells also contribute to the peri-vascular mesenchyme in the umbilical cord^23^, suggesting that *Sall4* and *Tbx4* are co-expressed in the precursor of umbilical cord. To address this possibility we compared expression patterns of *Sall4* and *Tbx4*. *Sall4* is expressed in the proximal part of the allantois at E8.5, while *Tbx4* is more broadly expressed in the allantois (Fig. 6i–l). Therefore, the expression patterns of *Sall4* and *Tbx4* overlap at the proximal part of the allantois. To further clarify their co-expression, we performed fluorescent *in situ* hybridization of *Tbx4* in combination with SALL4 immunofluorescence on sections of the E8.5 allantois. We detected nuclear SALL4 signals associated with cytoplasmic *Tbx4* mRNA signals in the allantois (Fig. m–q). These results demonstrate that cells expressing *Sall4* and *Tbx4* in the proximal allantois contribute to peri-vascular mesenchyme tissue in the umbilical cord.

### *Sall4 GCE* labels spermatogonia and spermatogonial stem cells in the postnatal testis

In the testis SALL4 is expressed mostly in undifferentiated spermatogonia, including spermatogonial stem cells (SSCs)^17–19^. To determine whether *Sall4 GCE* is active in undifferentiated spermatogonia and SSCs, we injected neonatal mice with tamoxifen and monitored CreER activity using the *R26-tdTomato* transgene at 7 and 60 days post tamoxifen injection. The earlier time point allowed identification of the initial labeled germ cell population and the later time point allowed identification of SSCs, which persist long-term. We used anti-red fluorescent protein (RFP) antibody to visualize tdTomato-positive cells.

**Figure 7:**
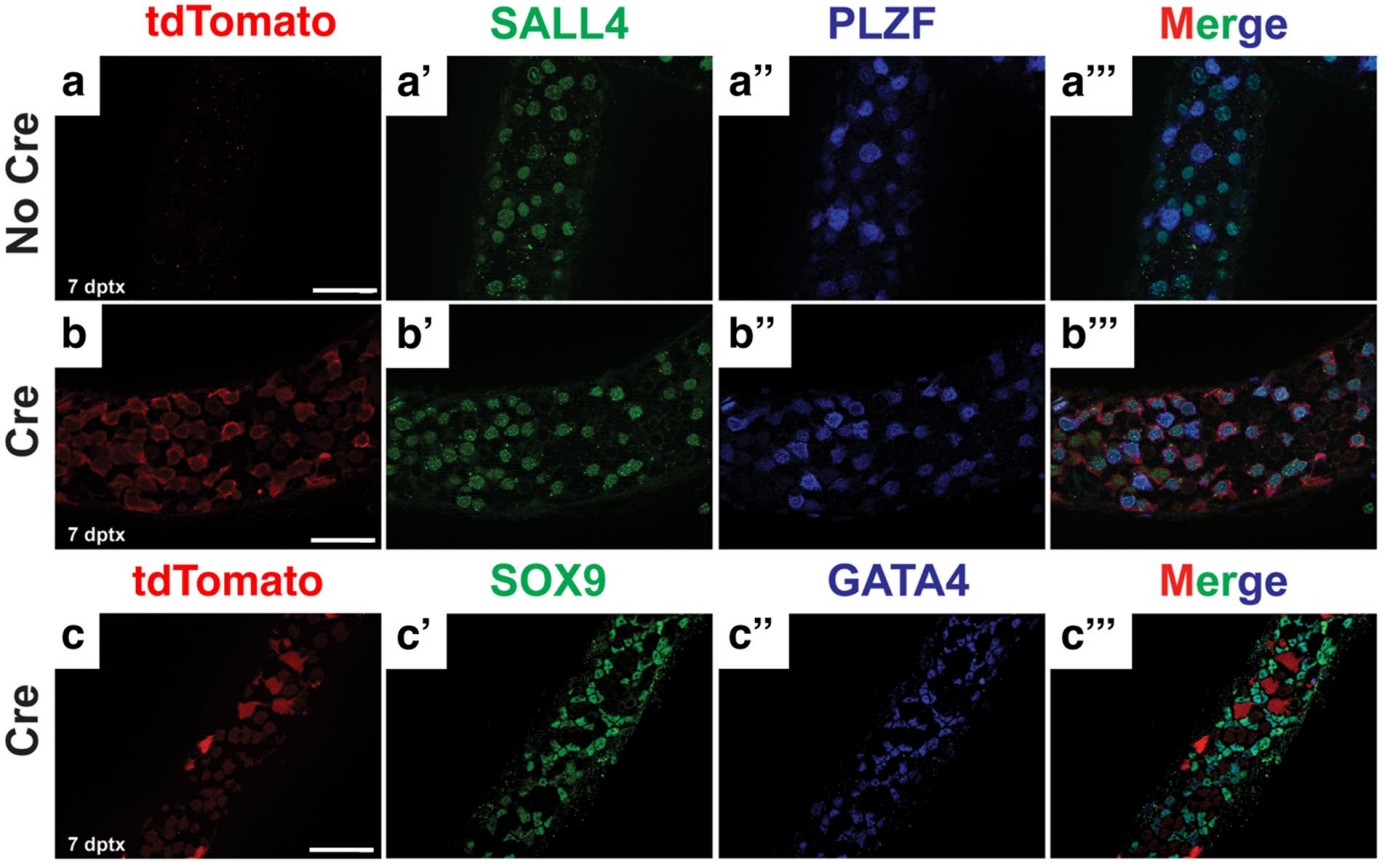
*Sall4 GCE* is active in undifferentiated spermatogonia. Immunofluorescence of wholemount seminiferous tubules. (**a**–a’”) *Rosa26-tdTomato* and (**b**–b’”,**c**–c’”) *Sall4 GCE; R26-tdTomato* testis 7 days post-tamoxifen treatment stained for tdTomato (red), SALL4 (green), SOX9 (green), PLZF (blue), GATA4 (blue). Scale bars in panels a,b,c: 100 µm. All panels are in the same scale.

To identify undifferentiated spermatogonia, we performed immunofluorescence on whole-mount seminiferous tubules from control and experimental mice 7 days post tamoxifen injection using anti-SALL4 and anti-PLZF antibodies. In controls lacking the *GCE* transgene, SALL4- and PLZF-positive cells were negative for tdTomato, and no tdTomato expression was detected in the entire gonad (Fig. 7a–a’”). In experimental animals, all SALL4- and PLZF-positive cells were positive for tdTomato (Fig. 7b–b’”). We also confirmed that the expression of tdTomato was limited to germ cells by co-staining tdTomato with the Sertoli cell markers SOX9 and GATA4 (Fig. 7c–c’”). Together, our data indicate that *Sall4 GCE* is active in undifferentiated spermatogonia, but not in Sertoli cells.

**Figure 8:**
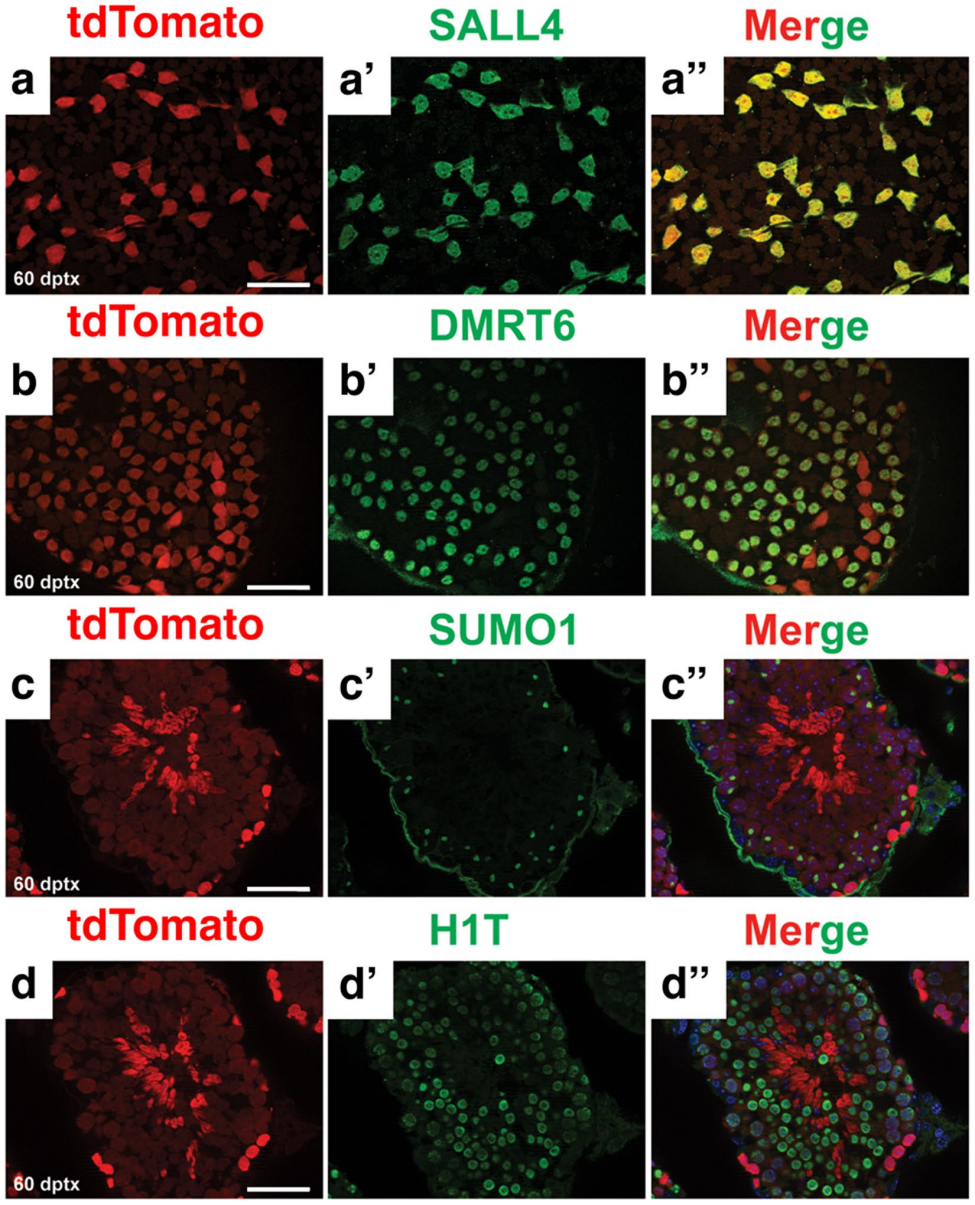
*Sall4 GCE* is active in SSCs. Immunofluorescence of wholemount (**a**–b”) and cross sections (**c**–d”) of seminiferous tubules. *Sall4 GCE; R26-tdTomato* testis 60 days post-tamoxifen treatment stained for tdTomato (red), SALL4 (green), DMRT6 (green), SUMO1 (green) and H1T (green). Scale bars in panel a,b,c,d: 100 µm. All panels are in the same scale.

To assess the activity of *Sall4 GCE* in SSCs, we examined testes 60-days post-tamoxifen injection. A cycle of spermatogenesis takes 35–40 days to complete and allows SSCs to produce differentiated progeny that give rise to all germ cell types^24,25^. If *Sall4 GCE* is active in SSCs, most germ cell types will be positive for tdTomato 60-days post activation of CreER, importantly including undifferentiated spermatogonia. We co-stained the testis with anti-RFP along with anti-SALL4 and anti-DMRT6 to label undifferentiated and differentiating spermatogonia, respectively. All SALL4 and DMRT6-positive cells were positive for tdTomato (Fig. 8a–a”,b–b”). We also used anti-small ubiquitin-related modifier-1 (SUMO1) and anti-linker histone H1T to label spermatocytes. These meiotic germ cells were positive for tdTomato, although these differentiated germ cell types have weaker tdTomato expression relative to spermatogonia (Fig. 8c–c”,d–d”). Together, these data suggest that *Sall4 GCE* is active in SSCs, since tdTomato-positive undifferentiated spermatogonia persisted for 60 days even as differentiating cells formed and underwent further development.

## Discussion

In this study, we sought to determine contributions of *Sall4*-expressing cells during the period of post-gastrulation mouse development when *Sall4* mRNA expression rapidly declines. LacZ staining at E13.5 showed drastic restrictions of LacZ-positive regions in embryos with tamoxifen injection at E8.5–E12.5. In particular, *Sall4* lineage contributions to the axial level declined rapidly. Moreover, whole mount analysis showed reduction of *Sall4* lineage contributions during these stages in other tissues, including the limb, craniofacial structures, and external genitalia in the embryo. Such genetic labeling to detect contributions of labelled cells is a well established approach, and is particularly useful in the case that gene expression patterns rapidly change/decline in developing embryos. However, an important caveat to note is that tamoxifen must be converted into an active metabolite to induce activation of CreER protein. Consequently, there is 0.5–0.75 day lag from tamoxifen injection to CreER-dependent recombination^21^. Given that *Sall4* expression pattern changes in the stages we analyzed, the LacZ staining pattern includes a lag to the corresponding *Sall4* mRNA expression pattern. In addition to the LacZ signals, we also have to note the characteristics of the GFP signals from the *GCE* cassette. The GFP signals are detectable only in the cells/tissues where *Sall4* is highly expressed, compared to *Sall4* mRNA expression in this study and previous reports, suggesting that the GCE cassette is a less sensitive indicator of expression than *in situ* analysis^14–16^. This low intensity of the GFP signals may be derived from the engineered *GCE* cassette. Although the GFP reporter signal is low, its expression can still act as a useful tool to detect and/or isolate high levels of *Sall4*-expressing cells.

Around E8.0–8.5, the forelimb field is determined and *Tbx5*, a marker of forelimb progenitors, starts its expression in the lateral plate mesoderm^26,27^. This timing of forelimb progenitor specification is correlated with the rapid decline of the contribution of *Sall4* lineages to the forelimb between E7.5 and E8.5. In hindlimbs, we found that LacZ signal intensity declined when tamoxifen was injected around the stage of hindlimb progenitor specification (E9.5). The temporal difference of reduction of *Sall4* lineage contribution to forelimbs and hindlimbs seems to involve differences in developmental timing between two types of limbs during body elongation: the broad contributions of *Sall4* lineages to hindlimbs persisted until E10.5, while the LacZ signal intensity gradually declined from E7.5–E10.5. As a consequence, the *Sall4* lineage contribution seems to be more significant in hindlimbs than forelimbs. Our previous study showed that conditional inactivation of *Sall4* by *TCre* caused subtle defects in forelimbs, while hindlimbs exhibited severe skeletal defects^15^. The rapid decline of *Sall4* lineage contribution to the forelimb and continued contribution to hindlimbs during E8.5–E9.5 provides a likely reason for this phenotypic difference.

Another interesting aspect with respect to *Sall4* conditional knockout phenotypes is that the *Sall4* lineage at E11.5 contributes to the anterior zeugopod and most anterior digit in hindlimbs. These elements are defective in *Hoxb6Cre; Sall4* conditional knockout hindlimbs, in which *Sall4* inactivation occurs later than that by *TCre*^15^. Interestingly, human patients with SALL4 heterozygous mutations also exhibit defects in the anterior limb elements. However, most of human patients exhibit limb defects primarily in forelimbs, with just a few cases in hindlimbs^3,28^. Anterior skeletal defects and *Sall4* contribution to the anterior part of the limb at E11.5 support the role of *Sall4* in formation of the anterior limb skeletal elements. A major unanswered question is the difference in limb types affected in human patients with *SALL4* heterozygous mutations versus mouse models of *Sall4* conditional knockout. The duration of *Sall4* lineage contribution to forelimbs and hindlimbs in this study correlates well with the severity of limb defects in *Sall4* conditional knockouts in our previous study^15^. Therefore, one possibility is that *SALL4*-expressing cells might contribute longer to forelimb buds than to hindlimb buds in human embryos. Studies of post-implantation human developmental processes, which may be obtained by differentiation of pluripotent stem cells *in vitro*, could help clarify the difference of limb phenotypes between human patients and mouse mutants.

It has been reported that several patients with SALL4 mutations also exhibit distinctive facial appearances. For instance, hypertelorism (increased distance between the eyes) as well as epicanthic folds that could be caused by failure of the nasal bridge to mature, have been reported^29^. Our observation of *Sall4* lineage contributions to the nasal structure is consistent with these phenotypes. Hemifacial microsomia, in which the lower half of one side of the face is underdeveloped, has also been reported in patients with SALL4 mutations^30^ and could be caused by defects in the branchial arch. Our finding that *Sall4* lineages contribute to the craniofacial region suggests that functional studies in mouse models, such as cell type-specific inducible knockout of *Sall4*, in the future would likely identify more detailed functions of *Sall4* in the craniofacial development, and these might be relevant to defects in human patients with *SALL4* mutations.

Neural tube closure defects, including exencephaly, are caused by complex genetic mechanisms^31,32^. A previous study showed that mutations in *Sall4* synergize with *Sall2* mutations to cause exencephaly in mouse embryos^33^. Interestingly, *Sall4* lineage contribution to the midbrain persisted even when tamoxifen was given at E10.5, when endogenous *Sall4* mRNA expression is low. This observation suggests that *Sall4* expressing cells continue to proliferate in the midbrain. The *Sall4* lineage contribution in the midbrain was observed as stronger LacZ signals with broader LacZ-positive domain when embryos exhibited an exencephaly phenotype. Such a LacZ staining pattern in exencephaly embryos suggests that over-proliferation of *Sall4*-expressing cells in the *Sall4* heterozygous genotype may contribute to exencephaly, in addition to functional interaction with *Sall2*.

*Sall4* lineage analysis in this study identified novel contribution of *Sall4* lineages to the extra-embryonic tissues, such as the yolk sac and the peri-vascular tissue in the umbilical cord. This finding led to identification of previously unappreciated expression of *Sall4* in the yolk sac and allantois. Interestingly, a previous genetic lineage tracing experiment led to the finding that *Tbx4*-expressing cells also contribute to the umbilical cord^23^. Both *Sall4* lineages and *Tbx4* lineages contribute to the mesenchyme of the cord but not to the endothelium. Moreover, both *Sall4* lineages and *Tbx4* lineages contribute to the external genitalia. These similarities suggest that *Sall4* lineages and *Tbx4* lineages share a common origin. In another extra-embryonic tissue, yolk sac, *Sall4* lineages also do not contribute to the endothelium. These observations highlight the distinct origin of the endothelium in the extraembryonic tissue.

Although *Sall4* expression in the genital primordium in mouse embryos was reported previously^14^, dynamic changes of *Sall4* lineage contribution to the external genitalia are a novel finding of this study. Restriction of *Sall4* lineage contribution to the posterior of the genital primordium during E10.5 and E12.5 suggests that roles of *Sall4* in the external genital primordium change during these stages. Sex determination in mice occurs around E10.5 when the *Sry* gene on the Y chromosome is briefly expressed^34^. However, the morphological differences of the external genitalia between males and females do not become evident until E16.5^35,36^. Our analysis of *Sall4* lineage contribution was at E13.5, before these differences appear. Studying *Sall4* lineage contributions to later stages could provide further insights into the roles of *Sall4* in genital organ development, including those specific to the male or female.

*Sall4* is expressed in spermatogonia and plays a role in SSC maintenance and spermatogonial differentiation^17,20^. By short (7 days) and long (60 days) lineage tracing in the testis, we found that *Sall4*-expressing cells contributed to not only undifferentiated spermatogonia but also differentiating spermatogonia and spermatocytes, confirming that *Sall4* expression marks spermatogonial stem cells. The lineage tracing also revealed that the contribution of *Sall4*-expressing cells is specific to germ cells. *Sall4* is expressed in all stages of undifferentiated spermatogonia, including A-single, A-paired and A-aligned spermatogonia^18^. Therefore, *Sall4 GCE* could be a tool to induce recombination in all these stages of spermatogonia.

In this study, we investigated *Sall4* lineage contribution to mid-gestation mouse embryos mostly by whole-mount LacZ staining. In addition to the tissues and organs characterized in this study, human patients with *SALL4* mutations exhibit symptoms in the heart, kidney and inner ear^3–7^. Detailed histological examinations of *Sall4* lineage in sectioned samples could help provide insights into roles of *Sall4* in these tissues and organs in the future. Moreover, as shown in the germ cells in the testis, the *Sall4 GCE* mouse line serves as an efficient lineage tracing tool in the postnatal mice. Therefore, *Sall4 GCE* could be used to determine contributions of *Sall4* lineages and elucidate roles of *Sall4* in the postnatal mice, providing detailed histological insights into the *Sall4* lineage contributions in the adult human body.

## Methods

### Generation of *Sall4 GCE* line

Knocking in the *GFP-CreER*^*T2*^ *(GCE)-Neo* cassette to replace the 1^st^ exon of the *Sall4* in C57BL/6 mouse embryonic stem cells and chimera production by blastocyst-injection were done at the University of Rochester Medical Center. The chimeric mice were bred with B6 albino, and germline transmission was confirmed by genomic PCR for Cre. The *Sall4 GCE-Neo* mice were bred with the *PGK-FLPo* mouse line (*B6.Cg-Tg(Pgk1-flpo)10Sykr/J*)^37^ to eliminate the *Neo* cassette. The *Sall4 GCE* mouse line is maintained on the C57BL/6 background.

### Breeding with reporter lines and tamoxifen injection

The *Sall4 GCE* mice were bred with *R26-LacZ* (*Gt(ROSA)26Sor*^*tm1S*^*°*^*r*^)^38^ or *R26-tdTomato* (*Gt(ROSA)26Sortm14(CAG-tdTomato)*^*Hze*^)^39^ reporter mouse lines. Noon of the day that vaginal plug is found is referred as embryonic day (E) 0.5. Tamoxifen (10 mg/ml, 100 µl) was administered to pregnant mice by intraperitoneal injection^40^. To neonatal pups, tamoxifen (10 mg/ml, 100 µl) was injected under the back skin on the day of birth.

### Whole-mount *in situ* hybridization and LacZ staining

Whole-mount *in situ* hybridization and whole mount LacZ staining on embryos were performed according to published procedures^15,41^. More than 10 embryos/stage were examined for *in situ* hybridization. For LacZ staining, 3–8 embryos were examined for each experimental setting of tamoxifen injection and LacZ staining.

### Immunofluorescence of yolk sac and umbilical cord

For whole mount immunofluorescence analysis of yolk sac, the yolk sac was fixed in 4% paraformaldehyde overnight at 4 °C, washed with phosphate buffered saline (PBS), and dehydrated with methanol. After rehydration, the yolk sac was blocked with 5% donkey serum in PBS + 0.1% Triton X-100, incubated with primary antibodies, washed and incubated with secondary antibodies. After washing, yolk sac was mounted on glass slides with 4′,6-diamidino-2-phenylindole (DAPI) fluoromount-G. For umbilical cord, the tissues were fixed, washed, dehydrated and rehydrated, and then cryosectioned at 14 µm. The slides were stained similar to the yolk sac samples. Fluorescent images were acquired with Zeiss LSM710 confocal microscopy. Primary antibodies used are shown in Supplementary Table 1. Alexa fluorophore-conjugated secondary antibodies (Invitrogen, 1:500) were used as secondary antibodies. Two samples were examined for both yolk sac and umbilical cord.

### Double detection of *Tbx4* mRNA and SALL4 immunoreactivities in the allantois

SALL4 immunofluorescence in combination with *Tbx4* mRNA fluorescent *in situ* hybridization was performed on cryo-sections of E8.5 allantois with modifications to our previously reported method^42^. Blocking of endogenous peroxidase (POD) was done with 3% hydrogen peroxide in PBS at room temperature for 30 minutes. Section *in situ* hybridization was performed using digoxigenin (DIG)-labelled *Tbx4* probe. After blocking with 10% heat-inactivated sheep serum, the slides were treated with anti-DIG-POD (Roche, Cat# 11207733910, 1:1000 dilution), and the signals were developed by the Alexa 488 Tyramid reagent (Invitrogen, Cat# B40953) by incubating the slides for 1 hour at 4 °C followed by 2 hours at room temperature. Then, the slides were incubated with anti-SALL4 antibody at 4 °C overnight, and the SALL4 signals were detected by Alexa594 goat anti-mouse IgG (Invitrogen, A-11005, 1:500). Sections from two embryos were examined.

### Immunofluorescence of testis sections

The testis was fixed in 4% paraformaldehyde overnight at 4 °C. After washing with PBS, the testes were processed to prepare 5 µm paraffin sections. Slides with paraffin sections were rehydrated and boiled with 10 mM of citric acid (pH 6.0). Slides were blocked with 10% serum (goat or donkey depending on the secondary antibody used) in PBS + 0.1% Triton X-100 at room temperature for 1 h and incubated with primary antibody (Supplementary Table 1) overnight at room temperature. The slides were washed, followed by 2 h incubation with secondary antibody. After washing, nuclei were stained with DAPI, and fluorescent images were captured with a Zeiss Imager Z1 microscope using a Zeiss MRm camera. Two testes were examined at P60.

### Immunofluorescence of whole mount seminiferous tubules

Whole mount immunofluorescence of seminiferous tubules was performed as previously described^43^. All images were captured with a Zeiss Imager Z1 microscope using a Zeiss MRm camera. Two testes at P7 and two testes at P60 were examined for this study.

### Experimental methods guideline statement

Animal experiments were performed according to the approval by the Institutional Animal Care and Use Committee of the University of Minnesota. Methods were carried out in accordance with relevant guidelines and regulations.

## Electronic supplementary material

Supplementary information

## Data Availability

Data generated or analyzed during this study are included in this published article. Additional datasets generated and analyzed during the study are available from the corresponding author on reasonable request.
