# Supplementary material for: Temporal changes of Sall4 lineage contribution in developing embryos and the contribution of Sall4-lineages to postnatal germ cells in mice
Source: Sci Rep. 2018 Nov 6;8:16410. doi: 10.1038/s41598-018-34745-5 (PMC6219540; doi:10.1038/s41598-018-34745-5)
Supplement: Supplementary file 1 — Supplementary information [file 41598_2018_34745_MOESM1_ESM.pdf]

Supplementary Information

Temporal changes of *Sall4* lineage contribution in developing embryos and the contribution of *Sall4*-lineages to postnatal germ cells in mice

Naoyuki Tahara, Hiroko Kawakami, Teng Zhang, David Zarkower and Yasuhiko Kawakami

Supplementary Table 1 Primary antibodies used for immunofluorescence

Primary Antibody		Dilution	Source	Reference
DMRT6	Rabbit polyclonal	1:100	Dr. David Zarkower	Zhang et al. PMID: 25249458
H1T	Rabbit polyclonal	1:200	Abacm	Cat. No. ab81498
GATA4	Goat polyclonal (IgG)	1:100	Santa Cruz Biotechnology	Cat. No. SC-1237 (C-20)
mCherry	Chick polyclonal (IgY)	1:500	Novus Biologicals	Cat. No. NBP2-25158
PECAM1	Goat polyclonal (IgG)	1:500	R&D Systems	af3628
PLZF	Goat polyclonal (IgG)	1:100	R&D Systems	Cat. No. AF2944
RFP	Rabbit polyclonal (IgG)	1:50	Rockland	Cat. No. 600-401-379
SALL4	Mouse monoclonal (IgG_1)	1:200	Santa Cruz Biotechnology	Cat. No. sc-101147 (EE-30)
SOX9	Rabbit polyclonal	1:200	MilliporeSigma	AB5535
SUMO-1	Mouse monoclonal (IgG_1)	1:200	Zymed Laboratories	Cat. No. 33-2400
TER119	Rat monoclonal (IgG_{2b})	1:100	BioLegend	Cat. No. 116201
VEGFR2	Rabbit monoclonal (IgG)	1:500	Cell Signaling Technology	Cat. No. 2479

Supplementary Table 2 List of non-standard abbreviations

DAPI	4',6-diamidino-2-phenylindole
DIG	digoxigenin
E	embryonic day
GCE	GFP-CreER^{T2}
P	Postnatal day
PBS	phosphate buffered saline
POD	peroxidase
RFP	red fluorescent protein
SSC	spermatogonial stem cells
SUMO1	small ubiquitin-related modifier-1
